# Effective Antimicrobial Activity of Green ZnO Nano Particles of *Catharanthus roseus*

**DOI:** 10.3389/fmicb.2018.02030

**Published:** 2018-09-03

**Authors:** Monika Gupta, Rajesh S. Tomar, Shuchi Kaushik, Raghvendra K. Mishra, Divakar Sharma

**Affiliations:** ^1^Amity Institute of Biotechnology, Amity University, Gwalior, India; ^2^Department of Biochemistry, National JALMA Institute for Leprosy and Other Mycobacterial Diseases, Agra, India; ^3^Interdisciplinary Biotechnology Unit, Aligarh Muslim University, Aligarh, India

**Keywords:** zinc oxide nanoparticles, biosynthesis, characterization, SEM, Catharanthus rouses, antimicrobial activity

## Abstract

In the present study, zinc oxide nanoparticles (ZnO NPs) were synthesized using leaf extract of *Catharanthus roseus* (*C. roseus*) under different physical parameters. Biosynthesis of ZnO NPs was confirmed by UV-Visible spectrophotometer and further, characterized by X-Ray Diffraction (XRD), Fourier Transform Infrared spectroscopy (FTIR), Scanning Electron Microscopy (SEM), Transmission Electron Microscopy (TEM), Energy-Dispersive X-ray spectroscopy (EDX), Atomic Force Microscopy (AFM), Photoluminescence study and Dynamic Light Scattering (DLS). We have also confirmed that several physical parameters such as pH, temperature, concentration of metal ions and reaction time were able to regulate shape and size of synthesized ZnO NPs. XRD and TEM analysis provided the information about the average size and hexagonal morphology of ZnO NPs. FTIR spectra analysis suggested that phenolic compounds played crucial role in the biosynthesis of ZnO NPs. The significant antibacterial activity of ZnO NPs was observed against *Staphylococcus aureus* MTCC 9760 (*S. aureus*), *Streptococcus pyogenes* MTCC 1926 (*S. pyogenes*), *Bacillus cereus* MTCC 430 (*B. cereus*), *Pseudomonas aeruginosa* MTCC 424 *(P. aeruginosa)*, *Proteus mirabilis* MTCC 3310 *(P. mirabilis)* and *Escherichia coli* MTCC 40 (*E. coli*). The synthesized ZnO NPs have shown antibacterial efficacy against both Gram-positive and Gram-negative pathogens. Synergistic effects of ZnO NPs and streptomycin showed increased efficacy as indicated by the increased zone of clearance in comparison to their individual effects (either ZnO NPs or streptomycin). Overall, the results elucidated a rapid, cost-effective, environmentally friendly and convenient method for ZnO NPs synthesis, which could be used as a potential antimicrobial agent against drug resistant microbes.

## Introduction

Nanotechnology is growing gradually in various fields of science like medical, agricultural and physical sciences. The production of nanoparticles (NPs) for the manufacturing of new smart materials at the nanoscale with unique properties has rapidly increased in the past few years (Albrecht et al., 2006; Singh et al., 2016). Nowadays, synthesis of metal NPs is an interesting area in nanoscience. Various types of metal NPs, like iron oxide, silver nitrate, copper oxide and zinc oxide have gained the attention of several research groups in the last few decades (Rouhi et al., 2013; Tiwari et al., 2018). There are several methods used for the synthesis of metal NPs like sol-gel method, thermal decomposition, hydrothermal, microwave irradiation and many more (Kolekar et al., 2013). However, these chemical and physical synthesis methods are time-consuming, costly and toxic due to the generation of a huge amount of secondary waste materials resulting from the addition of chemical agents for the reduction process.

Therefore, biological synthesis of NPs will be an option to reduce the toxicity, cost and time. Biological synthesis of NPs from plants extracts have elucidated as a better alternative as compared to physical and chemical methods. Biologically synthesized NPs are believed to be biocompatible, non-toxic and are used as drug carriers and fillings in medical materials (Rosi and Mirkin, 2005). Several studies based on green synthesis of ZnO NPs of various plants extract have exists, such as *Cassia tora* L. (Manokari and Shekhawat, 2017), *Sageretia thea* (Khalil et al., 2017), *Calotropis gigantean* (Chaudhuri and Malodia, 2017), *Azadirachta indica* (Bhuyan et al., 2015), *Hibiscus rosa-sinensis* (Bala et al., 2015), *Ocimum basilicum* L. *var. Purpurascens* (Bi et al., 2017), *Corymbia citriodora* (Zheng et al., 2015), *Zingiber officinale* (Raj and Jayalakshmy, 2015), and *Anisochilus carnosus* (Anbuvannan et al., 2015).

*Catharanthus roseus* belongs to the family Apocynaceae and known as Madagascar periwinkle, rose periwinkle, or rosy periwinkle and is a species of flowering plant family of Indian origin. It is a well-known medicinal plant and a rich source of secondary metabolites (Don, 1999) containing more than 200 terpenoid based indole alkaloids in various plant parts like leaf, stem, and root. These alkaloids are responsible for different medicinal properties like anticancer, astringent, anti-bacterial, anti-diabetic, anti-fungal, and anti-malarial activities (Noble, 1990). The reports published on the green synthesis of ZnO NPs using leaf extract of *C. roseus* are very few (Bhumi and Savithramma, 2014; Kalaiselvi et al., 2016) with fragmented knowledgebase. In the present study, green ZnO NPs were synthesized, characterized and their antimicrobial activities were evaluated. Synergistic effect of the synthesized NPs with pre-existing antibiotics was also observed during the study.

In the present study, *C. roseus* leaf extract was used for the biosynthesis of ZnO NPs under different physical conditions. The synthesized ZnO NPs were characterized through UV–visible spectroscopy followed by DLS (Dynamic Light Scattering), SEM (Scanning Electron Microscopy), EDX (Energy Dispersive X-ray analysis), TEM (Transmission Electron Microscopy), FTIR (Fourier Transform Infrared Spectroscopy), and XRD (X-Ray Diffraction). The antimicrobial properties of biosynthesized NPs were also evaluated against a number of gram-positive and gram-negative pathogenic strains, which suggested that these could be used as an alternative therapeutics against the drug-resistant microbes in the global emergence of drug resistance (Aderibigbe, 2017).

## Materials and Methods

### Preparation of Extract

*Catharanthus roseus* leaves powder prepared from the dried grounded leaves, collected from LNIPE, Gwalior (M.P.) India and used for NP synthesis. 6g of dried powder was mixed in 50 ml of distilled water and incubated at room temperature for 24 h. The extract was filtered and centrifuged for 30 min at 4000 rpm (Nagaonkar and Rai, 2015). The supernatant was used for ZnO NPs biosynthesis and stored at 4°C for further use.

### Biosynthesis of ZnO NPs

Biosynthesis of ZnO NPs was performed by mixing the 50 ml of aqueous solution of 0.01M zinc acetate dehydrate with 1 ml *C. roseus* leaf extract followed by constant stirring till the formation of the white suspension (Gnanasangeetha and Thambavani, 2013). The pH was adjusted to 12.0 using 2M NaOH and keep on stirring until the ZnO NPs precipitate were completely dissolved. The spectra exhibited an absorption band with a resolution of 1.0 nm between 350 and 500 nm (Jamdagni et al., 2016), this indicated the formation of ZnO NPs.

### Optimization of Various Physicochemical Parameters for ZnO NPs Biosynthesis

As per the report by Jamdagni et al. (2016) that physicochemical parameters regulate the green synthesis of inorganic metal NPs, several physicochemical parameters were optimized including pH, temperature; reaction time and concentration of metal ion.

### Effect of pH

pH was maintained at 8.0, 10.0, 12.0, and 14.0 by using 2M NaOH. The concentration of reaction mixture, the reaction time and temperature were kept constant. The absorbance of the solution was measured by the spectrophotometric approach. The procedure was repeated thrice to optimize the pH.

### Effect of Temperature

One of the important physicochemical parameter, temperature, was maintained at 30, 60, and 90°C using a water bath. The absorbance of the solution was measured by the spectrophotometric method as described earlier (Jamdagni et al., 2016). The concentrations of metal ions, the reaction time and pH of reaction mixture were kept as constant.

### Effect of Concentration of Metal Ion

The concentration of metal ion was maintained at 0.01M, 0.005M, and 0.0025M. The concentration of leaves extract, pH, temperature and incubation time of the solution were kept constant. The absorbance of the solution was measured at 300–500 nm as described above (Jamdagni et al., 2016).

### Effect of Reaction time

The stability of the solution was determined at room temperature at the interval of 0.5, 1, 2 h, and 4 h to study the effect of reaction time on the synthesis of NPs.

### Characterization of Metal Nanoparticles

ZnO NPs were characterized by several techniques *viz*., UV-Vis spectroscopy, FTIR, XRD, TEM, SEM, EDX and DLS, which provide important information for better understanding of the role of different physicochemical features.

### UV-Visible Spectroscopy

The bioreduction of zinc oxide was confirmed by subjecting diluted aliquots of the zinc metallic NPs to UV-Visible spectrophotometer (Model- Shimadzu UV- 2450, Japan) in the range of 300–500 nm (Talam et al., 2012).

### X-Ray Diffraction

The biologically reduced ZnO NPs solution was drop-coated on glass-slide. XRD measurement was carried out with the help of X-ray diffractometer (Model- Rigaku- miniFlex600). The pattern was recorded by Cu, K and α radiation with λ of 1.54 Å (Kumar et al., 2017). The particle sizes were calculated from the XRD pattern using Debye Sherrer’s method (D = 0.9λ/βcos𝜃). Where D is the crystallite size for the (h k l) plane, β is FWHM in radiations, θ is wavelength and 𝜃 is the XRD angle for the (h k l) plane (Chauhan and Mahajan, 2017).

### Fourier Transform Infrared Spectroscopy (FTIR)

The dried powder sample of ZnO NP was prepared by mixing purified NPs along with 10 mg potassium bromide (KBr) powder and then dried to remove the moisture content. Infrared spectra were recorded in the region of 500–4000 cm^-1^ on Perkin- Elmer FTIR-105627, United States (Mishra and Sharma, 2015).

### Particles Size Distribution

Particle size distribution analysis of ZnO NPs was performed by Zetasizer using DLS method as described by Omar et al. (2014), in order to determine the zeta potential and polydispersity index of the green synthesized NPs (nono plus - Zetasizer).

### Photoluminescence and Atomic Force Microscopy

Photoluminescence emissions and excitation spectra were recorded at room temperature by the spectrophotometer (Model- Shimadzu RF-5301 PC) (Parganiha et al., 2015). The surface topology of synthesized ZnO NPs was obtained by AFM analysis. A thin film of ZnO NPs deposited on silica glass plate by dropping few drops of the ZnO NPs solution on the plate and then allowed to dry at 30°C for overnight. The deposited film on silica glass plate was scanned with AFM Model Ntegra Prima AFM (NT-MDT, Russia) for the determination of the size, as previously described (Femi et al., 2011).

### SEM, EDX, and TEM

Scanning electron microscopy captured the surface morphology and size of the ZnO NPs. A thin film of the prepared ZnO NPs was deposited on a carbon-coated copper grid by dropping few drops of the ZnO NPs and allowed to dry at 30°C under the mercury lamp for 15 min. The carbon coated-copper grids were scanned with SEM-EDX (JEOL Model-LV6490) according to the study of Kumar et al. (2013). Samples for the TEM studies were prepared by placing a drop of the aqueous suspension of particles on carbon-coated copper grids followed by solvent evaporation under vacuum. The exact morphology and size distribution of the synthesized zinc oxide NPs were characterized by TEM. The TEM images were captured using JEOL- 1011, Japan (Nagaonkar and Rai, 2015).

### Antibacterial Activity of ZnO NPs

The antibacterial potential of ZnO NPs was determined against six different pathogenic bacteria (*S. aureus* MTCC 9760, *S. pyogenes* MTCC 1926*, B. cereus* MTCC 430, *P. aeruginosa* MTCC 424, *P. mirabilis* MTCC 3310 *and E. coli* MTCC 40), by the standard disk diffusion method (Diao et al., 2013). In brief, Whatman filter paper no.1 disks containing 700, 900, and 1500 μg of ZnO NPs/disks were used for the assay. The overnight grown cultures of tested bacteria serially diluted to 1 × 10^-7^ colony forming unit (CFU) were used for the assay. The antibacterial activity of ZnO NPs was determined by measuring the diameter of zones of inhibition after 24 h of incubation at 37°C. The minimum inhibitory concentration (MIC) of ZnO NPs was determined by serial dilution method as discussed previously by Dobrucka and Dugaszewska, 2016. The MIC was determined against the serially diluted bacterial concentration of 10^-5^, 10^-6^, 10^-7^ CFU/ml along with different concentrations of ZnO NPs (700, 900, and 1500 μg/ml). The serially diluted bacterial cultures were incubated at 37°C and optical density (OD) was taken at 590 nm (Dobrucka and Dugaszewska, 2016).

### Synergistic Activity of ZnO NPs With Streptomycin

The synergistic antibacterial potential of zinc oxide NPs with streptomycin was determined against six pathogenic bacteria by the standard disk diffusion method (Rakholiya and Chanda, 2012; Diao et al., 2013). Different disks were prepared by using streptomycin 10 μg/ml with varying concentrations of ZnO NPs (700, 900, and 1500 μg/ml) and then used for testing the antibacterial activity on agar plate against the test microbes. After 24 h of incubation at 37°C, the synergistic antibacterial activity of the zinc oxide NPs and streptomycin mixture were measured in terms of the diameter of the zone of inhibition.

## Results and Discussion

### Optimization of Physical Parameters of ZnO NPs

Earlier studies investigated the green biosynthesis of ZnO NPs by using reducing and capping agents, which are stable and mono-dispersed (Bhumi and Savithramma, 2014; Kalaiselvi et al., 2016). In this study, *C. roseus* was found to be a good source for the green synthesis of ZnO NPs. The absorption peak was observed between 300 and 500 nm wavelength. It was reported previously that several external physical parameters are responsible for regulation of monodispersity of NPs (Bhumi and Savithramma, 2014; Kalaiselvi et al., 2016). In the biosynthesis of ZnO-NPs, zinc oxide was reduced by *C. roseus* leaf extract, which contains a number of secondary metabolites like vincristine, vinblastine and serpentine. Probably these secondary metabolites act as the stabilizer, reducing and capping agent. These properties of *C. roseus* provide narrow absorption band of zinc oxide NPs at 366 nm. The reduction process depends on the excitation of surface plasmon vibrations of the metal NPs. Previously it was reported that surface plasmon resonance (SPR) bands are influenced by several factors like the concentration, temperature, size and shape of synthesized NPs, pH etc. (Stepanov, 1997; Kelly et al., 2003).

### Effect of pH

**FIGURE 1:**
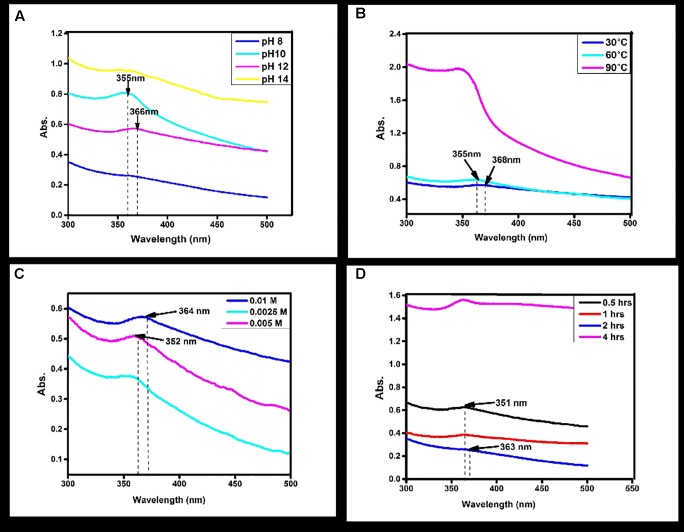
Normalized absorption spectra of Zinc oxide nanoparticles obtained **(A)** at different pH values of the reaction mixture using *Catharanthus roseus* leaf extract **(B)** and at different reaction temperatures using *Catharanthus roseus* leaf extract **(C)** at different Molarity of zinc acetate **(D)** at different time intervals of reaction time using biosynthesized zinc oxide nanoparticle.

To study the effect of pH, considered the most important parameters affecting the NPs formation, leaf extract (1.0 ml), bulk zinc acetate (0.01M), incubation time and temperature (room temperature) were kept constant. It was found that at pH 12, the characteristic absorption peak of ZnO NPs observed at 366 nm. At high pH 14 and lower pH 10-8, no absorption peaks were observed (**Figure 1A**). It is suggested that at pH 12, zinc acetate dihydrate was converted to ZnO NPs, which showed the metal oxide reduction and synthesis. Most NPs were spherical in shape at pH 12. Similar results were reported in the flower extract of Nyctanthes arbor-tristis (Jamdagni et al., 2016).

### Effect of Temperature

To see the effect of temperature as a second major controller on the synthesis of NPs other parameters were kept constant. Increase in the temperature from 60 and 90°C, shows no absorption peak (**Figure 1B**). At 30°C temperature, the absorption peaks observed at 366 nm which suggested zinc acetate was converted into ZnO NPs. The similar temperature was also used in synthesizing of ZnO NPs from the seed extract of *Murraya koenigii* (Sundaraselvan and Quine, 2017).

### Effect of Metal Ion Concentration

Synthesis of NPs is also affected by the concentration of zinc acetate dihydrates; the NPs formation was increased while increasing the concentration of zinc acetate dihydrates. In the present study different concentration (0.0025M, 0.005M, and 0.01M) were used for the synthesis of particles. By increasing the concentration of dihydrated zinc acetate from 0.0025M, 0.005M, and 0.01M were clearly demonstrated the effect of concentration on absorption peak. No absorption peaks were observed at 0.0025M and 0.005M (**Figure 1C**). Furthermore, it is clearly indicated that the absorption peak was observed at 0.01M by UV–Vis absorption peak and depicted that the dispersion of ZnO NPs was affected by the concentration. The characteristic peak 366 nm was recorded at 0.01M dihydrates zinc acetate. Similar results were observed with flower extract of *Nyctanthes arbor-tristis*, where increasing concentrations of zinc acetate (0.0025–0.01 M) were used to optimize the synthesis of ZnO NPs (Jamdagni et al., 2016).

### Effect of Reaction Time

For the synthesis of NPs, reaction time is also considered as an important factor. The reaction time depends on the nature of metals also. Reaction time is the period required for complete reduction of the metal ions for the synthesis of metal NPs. To study the effect of incubation time, the concentration of *C. roseus* leaves extract and dihydrate zinc acetate was kept constant. By increasing the reaction time from 0.5, 1, 2, and 4 h, results shows that no absorption peak was observed in 0.5, 1, and 4 h (**Figure 1D**). The absorption peak at 363 nm was observed at reaction time 2 h. Change in the reaction time may inhibit the formation of NPs. This is in close agreement with the previously published studies on *C. roseus*, where observation peak was also recorded at 3 h (Kalaiselvi et al., 2016).

### Fourier Transforms Infrared Spectroscopy (FTIR) Analysis

**FIGURE 2:**
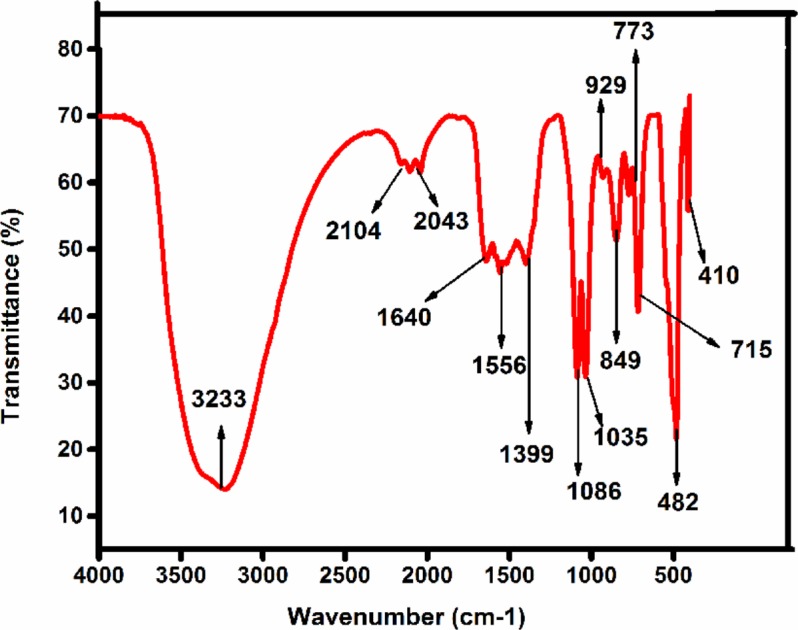
The FTIR spectra of synthesized Zinc oxide nanoparticles using *Catharanthus roseus* leaf extract were measured in % transmittance in the wave number frequency range of 4000-500 cm^-1^.

The FTIR spectrum showed (**Figure 2**) peaks at 3233, 2104, 1640, 1556, 1399, 1086, 926, 773, 849, 715, 1035, 482, 410 cm-1. The broad peak at 3233 cm-1 could be assigned to C-H stretch of an alkenyl group and 2104 cm-1 shifted to -C≡C- stretching vibrations (Yedurkar et al., 2016). The peak obtained at 1640 corresponded to C = O stretching of functional group, peak in the range 1556 corresponded to C = C / amine – NH stretching of the aromatic compound (Rastogi and Arunachalam, 2011). The peak at 1399 refers to C-H stretching vibrations of the alkene group. The peaks 1086 results from stretching amine, and five peaks at 929,773,849,715 1035 from C-N stretching amine group respectively (Valentina and Boris, 2013). FTIR peaks have functional groups displayed in **Table 1**. It is reported that secondary metabolites present in *C. roseus* could be responsible for the reduction of zinc acetate dihydrate into ZnO NPs. FTIR spectra peak represents a high-intensity broadband around 3233 cm^-1^ due to stretching alkenyl group made of zinc acetate salts and their reduction of ZnO NPs.

**Table 1:** Functional Group present in synthesis of ZnO NPs analyzed by FTIR.

S. no	Absorption peak (cm^-1^) in ZnO NPs	Bond/functional groups
1	3233	C-H stretch of alkenyl
2	2104	-C≡C- stretching
3	1640	C = O stretching
4	1556	C = C/amine – NH stretching
5	1399	C-H stretching
6	1086	stretching amine
7	929	C-N stretching amine
8	773	C-N stretching amine
9	849	C-N stretching amine
10	715	C-N stretching amine
11	1035	(C-O), C-F, alkyl halide strong


### Particle Size Distribution

**FIGURE 3:**
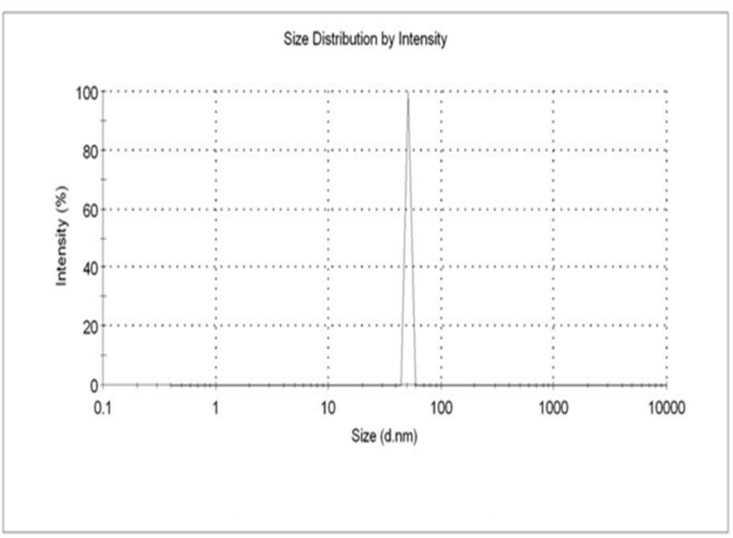
The DLS analysis of zinc oxide nanoparticles were maintained at pH 12 were kept at 30°C using continuous magnetic starrier for 2 h with 0.01M Zinc acetated Molarity.

Dynamic light scattering analysis suggested particles size of ZnO NPs showed the average size distribution in the range of 50.73 nm along with the polydispersity index 0.780. Samples with very broad size distribution have polydispersity index values > 0.7 (Nagarajan and Kuppusamy, 2013). **Figure 3** clearly indicates that the obtained ZnO NPs are polydispersible in nature. DLS is a commonly used method for particles size distribution in colloidal solution.

### Effect of Various Parameters on ZnO NPs Size Distribution

In the present study, the effects of different parameters on the particle size distribution of NPs were observed. Results were also compared with the variations in DLS data and peaks obtained from UV analysis.

**FIGURE 4:**
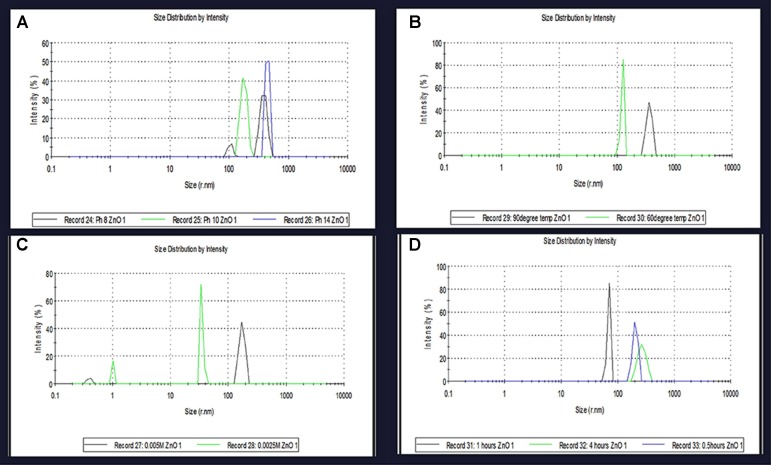
The particles size variatioparticles size variation at optimum parameters were confirmed by DLS analysis of zinc oxide nanoparticles using *Catharanthus roseus* leaf extract. **(A)** SPR peak shifted at different pH values depend on the particles size variation **(B)** at different reaction temperatures effect on particles size **(C)** biosynthesized zinc oxide nanoparticles were also effect on particles size at different Concentration of metal ion **(D)** and at different time intervals of reaction time using biosynthesized zinc oxide nanoparticle size were also effect on particles size and compare with SPR band.

At different pH, *viz*. 8, 10 and 14, diverse sizes of NPs were synthesized. The DLS data confirms variation in the size of NPs and its dependence on pH. The smallest size (50 nm) of the biosynthesized NPs was obtained at pH 12 while at pH 8 NPs of size 385 nm, at pH 10 NPs of size 178 nm and at pH 14 NPs of size 445 nm were observed (**Figure 4A**). At different temperatures 60 and 90°C, different sizes of NPs were synthesized. The DLS data confirmed that variations in size of NPs due to variation in temperature conditions. The obtained results depicted in **Figure 4B** showed that at 60°C NPs size was 124 nm, while at 90°C NPs size was 383 nm. The DLS data confirms variation in the size of NPs as a dependent factor on the concentration of metal ion. At different metal ion concentrations from 0.005M and 0.0025M, different sized NPs were synthesized. The smallest size of the NPs synthesized at 0.01M metal ion concentration was of 50 nm size. At 0.005M metal ion concentration NPs size was 173 nm, while at 0.0025M, 34.78 nm NPs size was observed. The DLS data confirm the variation in the size of NPs due to reaction time conditions. At different reaction time, i.e., at 4, 2, 1, and 0.5 h, NPs of different sizes were synthesized. The smallest sizes of the synthesized NPs at 2 h reaction time was 50 nm. The obtained results depicted in **Figure 4D** clearly showed that at 1 h of reaction time NPs of size 69.30 nm were synthesized, while at 0.5 h reaction time NPs of size 204 nm and 4 h reaction time NPs of size 269 nm were synthesized.

### Photoluminescence Spectra Analysis

**FIGURE 5:**
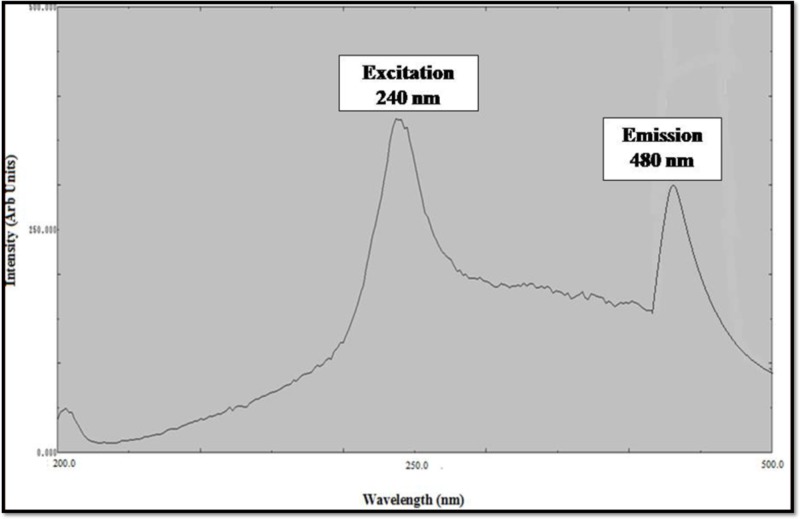
Excitation and Emission Spectra of zinc oxide nanoparticles using *Catharanthus roseus* leaf extract.

Photoluminescence spectrum of ZnO NPs synthesized from the leaf extract of *C. roseus* shows the presence of the peak at 280 nm in accordance to excitation of ZnO NPs and the peak at 480 nm is correspondence to strong ultraviolet emission (**Figure 5**). In the PL spectra, the luminescent region was detected between the wavelength of 280–480 nm, due to interstitial zinc and the presence of the acceptor and donor states in the region between the valence and conduction band. ZnO NPs show strong, visible emission due to the compartment of a huge number of open flaws. This is due to the decrease in ratio of surface to volume ratio of ZnO NPs. These results showed the large number of defects on the surface of NPs (Parganiha et al., 2015).

### Atomic Force Microscope Analysis

**FIGURE 6:**
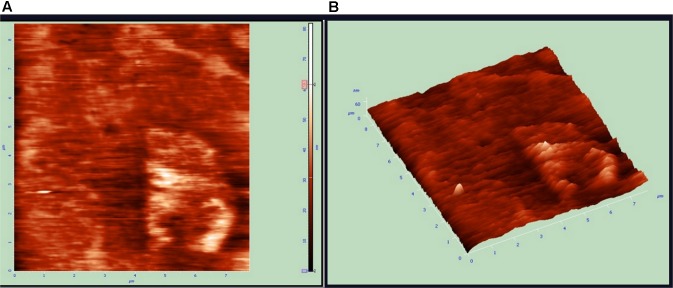
Atomic force microscopy (AFM) results of zinc oxide nanoparticles 2D and 3D Images. **(A)** Unfiltered AFM image showing topographical 2D image of Zinc oxide nanoparticles **(B)** 3D image of synthesized.

Atomic force microscopy (AFM) analysis is a commonly used technique for the determination of the size of NPs. AFM gives us insight about the roughness of ZnO NPs (Femi et al., 2011). The size of metal NPs was observed from tip-corrected AFM measurements and the shape of ZnO NPs were determined. The tip-corrected measured the size of NPs in the range of 50–82 nm (**Figure 6**). The result showed the 2D and 3D view of the sample surface over a 2 × 2 μm scan and uniform height distribution around 50 nm (**Figure 6**). Similarly, these results also showed the 2D AFM image of ZnO NPs using line profile. The result of line profile images of ZnO NPs depicted NPs of size 50 nm. Atomic force microscope analysis of ZnO NPs indicated that the changes in different parameters like temperature, pH, the concentration of metal ion and incubation time significantly affected the shape and size of the NPs. The increase in reaction temperature from 60 to 90°C greatly affected the particle size of ZnO NPs (**Figure 4B**). Similarly, variations in particle size distribution were found because of pH, the concentration of metal ion and incubation time (**Figures 4A,C,D**). The XRD results also confirmed NPs size (Rajendran and Kandasamy, 2017). The polydispersed ZnO NPs size measured by AFM 3D image was in the range of 50–82 nm.

### SEM and EDAX Analysis

**FIGURE 7:**
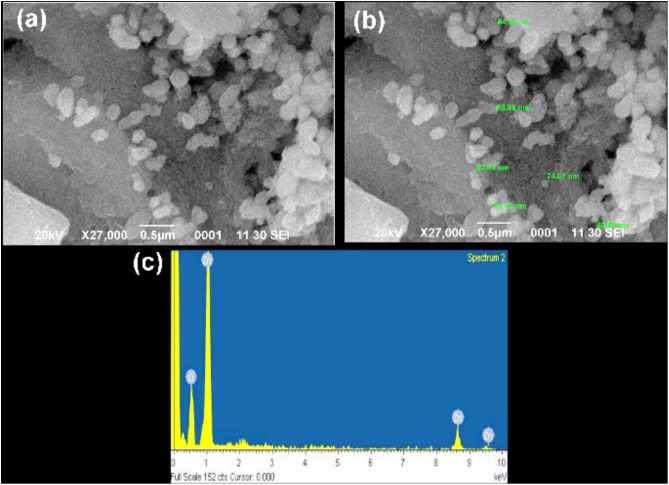
Scanning electron microscopy (SEM) images of the synthesized zinc oxide nanoparticles. **(a)** SEM images of Zinc nanoparticles, reveals its uniformity **(b)** image showed the sizes of nanoparticles within the range of 62–94 nm **(c)** EDX analysis of the zinc oxide nanoparticles strong signal of zinc and weak signal of oxides, which confirms the elemental composition of zinc oxide nanoparticles. In this co-large image no duplication of images exist.

Scanning electron microscopy also confirmed the morphology and size of NPs (Datta et al., 2017). The scanning electron microscope images of ZnO NPs showed size ranging between 62 and 94 nm (**Figures 7a,b**), which clearly demonstrated the presence of spherical shaped ZnO NPs. EDAX analysis of ZnO NPs; zinc (Zn) and oxygen (O) which clearly confirms the presence of metallic zinc acetate in the biosynthesis of ZnO NPs (Zheng et al., 2015). EDAX spectrum represents two peaks of zinc and oxygen each (**Figure 7c**). The EDAX analysis shows the optical absorption peaks due to SPR of ZnO NPs. The presence of oxygen in trace amount represents the involvement of plant phytochemical groups in reduction of metal ion, capping agents and stability of the biosynthesis of Zinc oxide NPs. The elemental profile of biosynthesized Zinc oxide using *C. roseus* leaf extract showed higher counts at 10 keV due to zinc oxide ions, which confirmed the formation of ZnO NPs.

### X-Ray Diffraction Analysis

**FIGURE 8:**
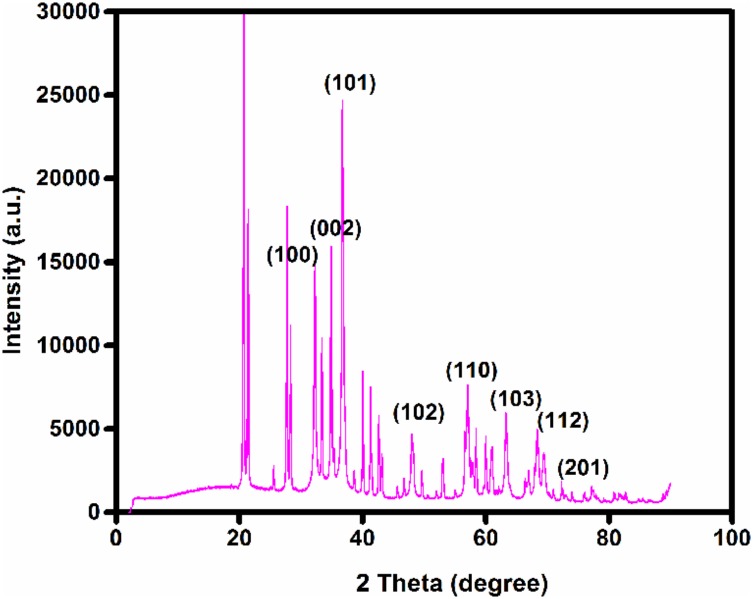
X-ray diffraction (XRD) patterns of zinc oxide nanoparticles obtained using *Catharanthus roseus* leaf extract as reducing agents.

Bragg’s reflection peaks observed by the XRD pattern (**Figure 8**) determined crystalline nature of ZnO NPs. The broadening of peaks clearly indicates the formation of ZnO NPs within the nanometer range. For ZnO, Bragg’s reflections peaks at 2𝜃 value of 34.88, 36.72, 47.99, 56.56, 63.68, 67.90, and 69.36° corresponds to (002), (101), (102), (110), (103), (112), and (201) lattice planes respectively (Rajendran and Kandasamy, 2017). These peaks were in good agreement with the reported literature and well consistent with the JCPDS file No. 89–1397 (Hamedani and Farzaneh, 2006). Thus showing that the synthesized NPs are identical to the hexagonal phase of zinc oxide. The XRD was recorded in the range of 20–80°, most of the diffraction peaks can be assigned to zinc oxide according to the report. The highest intensity peak (101) plane of zinc oxide with other smaller intensity peaks (002, 102, 110, 103, 112) was observed. **Table 2** represents the details of full-width half maxima (FWHM), Miller indices, d- spacing in nanometer, D - granule size of ZnO NPs (Parthasarathy et al., 2017). The Average particle size of Zinc oxide was 36.83 nm at (101) through Debye Sherrer’s formula. XRD pattern corresponding to impurity was absent. This XRD data thus proves the purity of synthesized ZnO NPs. There was concordance between our experimentally observed spacing data and calculated data.

**Table 2:** Particles size and simple peak indexing of zinc oxide nanoparticles.

2𝜃 of the intense peak (deg)	𝜃 of the intense peak (deg)	FWHM of intense peak (β) in radian	Size of the particle (D) nm	d-spacing nm	(h k l)
34.88	17.44	0.00308	47.20 nm	0.257 nm	002
36.72	18.36	0.00418	36.83 nm	0.244 nm	101
47.99	23.99	0.00849	36.24 nm	0.189 nm	102
56.56	28.28	0.00226	69.41 nm	0.162 *nm*	110
63.68	31.84	0.010819	15.09 nm	0.146 *nm*	103
67.90	33.95	0.00232	72.04 nm	0.137 nm	112
69.36	34.68	0.00844	19.98 nm	0.135 nm	201


### TEM Analysis

**FIGURE 9:**
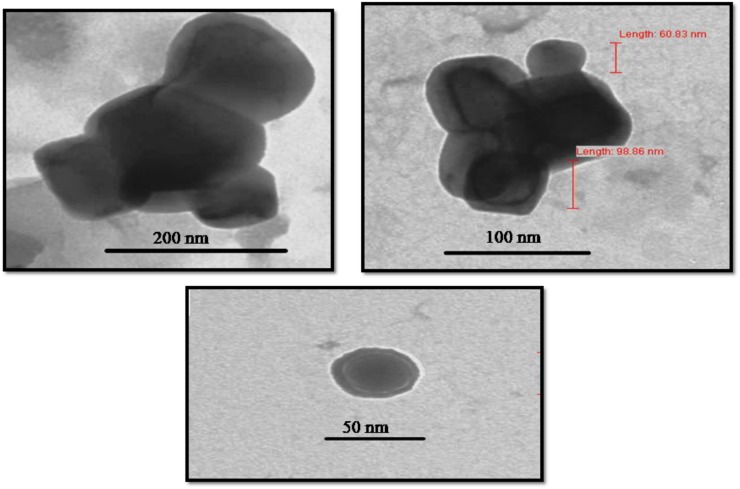
Morphological characterization of *Catharanthus roseus* leaf derived zinc oxide nanoparticles. The TEM image of ZnO NPs reveals its sizes as 50–92 nm.

Transmission electron microscopy images provided exact morphology and size of the synthesized zinc oxide NPs at different magnification (**Figure 9**). At pH 12, the hexagonal wurtzite shapes of ZnO NPs were observed with an average diameter ranging from 50 to 92 nm. The results were confirmed by DLS as well as compared with Scherrer analysis which also confirmed the size of synthesized NPs to be in the range of 50–90 nm (**Figure 3** and **Table 2**). Published literature (Nair et al., 2009; Salem et al., 2015) showed that smaller the size of ZnO NPs, greater is the efficacy in inhibiting the growth of bacteria (Farzana et al., 2017). The toxicity of ZnO NPs depends on the solubility, as the solubility increases, toxicity is also reported to be increased. Increased solubility leads to better accumulation of NPs on the bacterial cell surface and thus better antimicrobial activity (Datta et al., 2017).

### Antimicrobial Activity of ZnO Nanoparticles

The antimicrobial activity of ZnO NPs was demonstrated with the help of the disk diffusion method. The different concentration of NPs were tested against *S. aureus* MTCC 9760, *S. pyogenes* MTCC,1926 *B. cereus* MTCC 430, *P. aeruginosa* MTCC 424, *P. mirabilis* MTCC 3310 and *E. coli* MTCC 40 bacteria. The Zinc oxide NPs at 1500 μg/ml displayed good antibacterial activity against all the six pathogenic bacteria included in the study, as indicated by the diameter of inhibition zones of 11.09–11.74 mm as compared to streptomycin (**Table 3**). These results showed that ZnO NPs undergo an interaction with the bacterial cell. The results indicated better action against gram-positive bacteria as compared to gram-negative bacteria.

**Table 3:** Synergistic antibacterial activity of ZnONPs (700, 900, and 1500 μg/ml) with standard antibiotics streptomycin (10 μg/ml) against pathogenic bacteria.

Bacteria	Streptomycin (10 μg/ml)	Single concentration of ZnONPs 1500 μg/ml (10^-7^ CFU/ml)	ZnONPs + Streptomycin 700 μg/ml (10^-7^ CFU/ml)	ZnONPs + Streptomycin 900 μg/ml (10^-7^ CFU/ml)	ZnONPs + Streptomycin 1500 μg/ml (10^-7^ CFU/ml)
*P. aeruginosa*	11.03 ± 0.03	11.09 ± 0.01	11.22 ± 0.04	12.49 ± 0.11	13.86 ± 0.83
*E. coli*	10.07 ± 1.04	11.10 ± 0.03	11.42 ± 0.28	12.61 ± 0.74	12.52 ± 0.39
*P. mirabilis*	11.03 ± 0.90	11.14 ± 0.03	11.24 ± 0.19	11.61 ± 0.03	13.6 ± 0.51
*S. aureus*	11.15 ± 0.00	11.74 ± 0.03	12.06 ± 0.06	13.60 ± 0.02	15.61 ± 0.48
*S. pyogenes*	11.12 ± 0.01	11.52 ± 0.02	11.56 ± 0.01	12.25 ± 0.03	14.07 ± 0.03
*B. cereus*	11.12 ± 0.00	11.50 ± 0.00	11.51 ± 0.01	12.37 ± 0.19	14.17 ± 0.15


**FIGURE 10:**
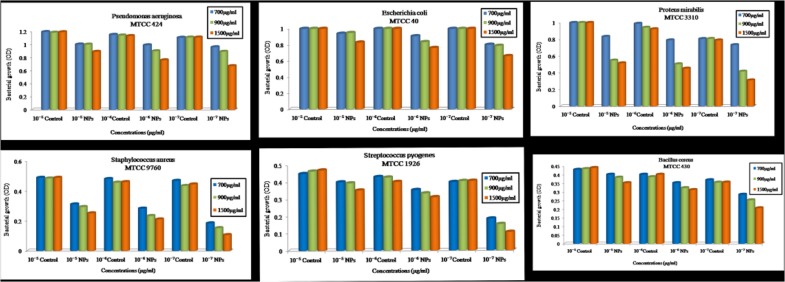
Optical density vs. Concentration of zinc oxide nanoparticles using *Catharanthus roseus* leaf extract were done by serially diluted of MIC assay at 700, 900, 1500 μg/ml concentration against six pathogens such as *Pseudomonas aeruginosa*, *Escherichia coli*, *Proteus mirabilis*, *Staphylococcus aureus*, *Streptococcus pyogenes*, and *Bacillus cereus.*

Effect of ZnO NPs on the bacterial growth curve was determined in the presence of ZnO NPs in the culture media having different bacterial cultures separately. Microorganisms were grown in (MHB) Muller Hinton broth medium until log phase. The antibacterial activity was determined against the serially diluted bacterial concentration of 10^-5^, 10^-6^, 10^-7^, CFU/ml with different concentrations of ZnO NPs (700, 900, and 1500 μg/ml). At 700 and 900μg/ml concentration of ZnO NPs, the bacterial growth was slightly diminished. Moreover, when the concentration of ZnO NPs was 1500 μg/ml, the growth of *Staphylococcus aureus* MTCC 9760*, Streptococcus pyogenes* MTCC 1926, *Bacillus cereus* MTCC 430, *Pseudomonas aeruginosa* MTCC 424, *Proteus mirabilis* MTCC 3310 and *Escherichia coli* MTCC 40 were completely inhibited (**Figure 10**).

Complete growth inhibition at higher concentrations of ZnO NPs was probably due to the inhibitory effect of biologically synthesized ZnO NPs on pathogenic bacterial growth. Therefore, we suggested that the zinc oxide is bacteriostatic at the low concentration while bactericidal at high concentration. These results are similar to earlier studies (Divya et al., 2013; Divyapriya et al., 2014).

### Synergistic Antimicrobial Effect of ZnO NPs

**FIGURE 11:**
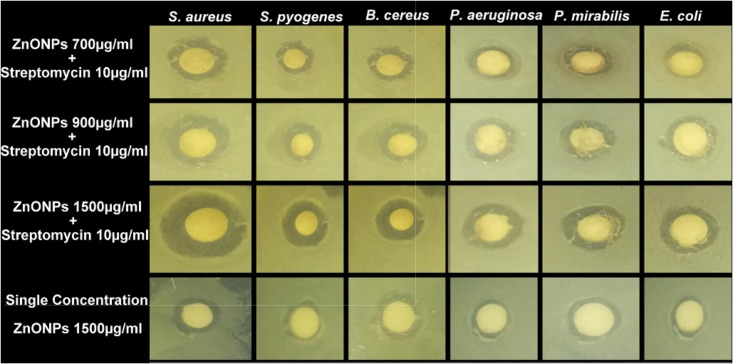
Antibacterial activity of ZnONPs (700, 900, and 1500 μg/ml) and synergistic antibacterial potential of ZnONPs mixed with standard antibiotics, streptomycin (10 μg/ml).

The synergistic effect of zinc oxide NPs and streptomycin was evaluated against six pathogenic bacteria and the results are shown in **Figure 11** and tabulated in **Table 3**. Streptomycin (10 μg/ml), alone did not show very good activity against the tested pathogens. Similar results were also obtained with zinc oxide NPs at 1500 μg/ml concentration. However, the combined effect of antibiotics and zinc oxide NPs displayed strong antibacterial activity against all the tested pathogens at 1500 μg/ml, with a zone of inhibition ranging in diameter from 12.52 to 15.61 mm (**Table 3**) as compared to 700 and 900 μg/ml. The maximum zone of inhibition was obtained against gram-positive bacteria at 1500 μg/ml concentration as compared to gram-negative bacteria (*Pseudomonas aeruginosa* MTCC 424, *Escherichia coli* MTCC 40, *Proteus mirabilis* MTCC 3310). Synergistic effect of ZnO NPs and streptomycin suggested its effectiveness against the test pathogens as a combinatorial therapy.

It was previously, reported that ZnO NPs have a broad spectrum of antibacterial activity. The antibacterial activity of ZnO NPs increases due to the reduction in particle size (Yamamoto, 2001). However, the exact mechanism of ZnO NPs against bacteria is still unknown. The proposed antibacterial activity may be because of following reasons: (i) direct or electrostatic interaction between ZnO NPs and cell membrane (ii) cellular internalization of ZnO NPs, (iii) production of active oxygen species. Based on these hypotheses, it is suggested that direct interaction of ZnO NPs with the bacterial cell surface, change the permeability of cell membrane toward the NPs. The NPs gain entry inside the cell and induce oxidative stress which causes inhibition of cell growth and finally cell death (Xie et al., 2011). Nowadays synergistic study becomes a very prominent tool to increase the antibacterial activity of existing antibiotics or available natural products. In the present study, the synergistic activity of ZnO NPs with the antibiotics was demonstrated. Due to the small size and easy penetration power of NPs along with antibiotics, their synergistic effect leads to the death of bacteria as these particles may pose serious damage to the vital machinery of bacteria. Previously several reports have been published on the synergistic potential of NPs with the antibiotics against a number of dreadful multi-drug resistant pathogenic bacteria (Jiménez et al., 2015). All these studies have proposed that a synergistic mechanism will decrease the use of high concentration dose of antibiotics against bacteria. Our results confirm the previous idea of using NPs in synergism with prescribed antibiotics so that the problem of increasing drug resistance among microbes can be tackled efficiently.

**FIGURE 12:**
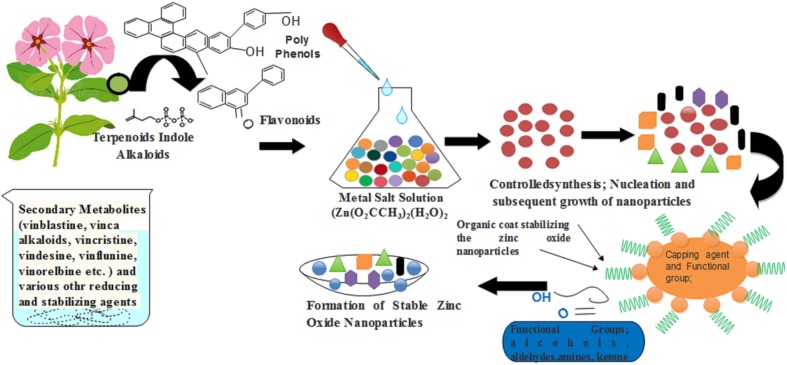
A schematic diagram that showed how the *C. roseus* help in the synthesis of green zinc oxide nanoparticles.

*Catharanthus roseus*, is a rich source of several medicinally important secondary metabolites including vinblastine, vincristine, alkaloids ajmalicine, serpentine, phenolic compounds etc. These compounds work in both capacities: (i) principle reducing agent for zinc oxide NPs synthesis process and the (ii) probable stabilizing agent. As discussed previously by Mayedwa et al. (2017); reported that plant-mediated synthesis could not be regulated by enzymes because usually the green natural extract is heated at high temperature during the synthesis process of NPs. According to them, phytochemicals such as phenolic, natural sterolin, terpenoids, and flavonoids are involved in reducing and act as capping agents of zinc oxide NPs (**Figure 12**).

## Conclusion

In the present study, ZnO NPs were synthesized by using the green approach as leaves extract of *C. roseus* at pH 12 was used for the synthesis process. The size of synthesized NPs was measured to be in the range of 50–92 nm. Further XRD, FTIR, SEM, TEM, EDX, AFM, and DLS techniques were used for the characterization of synthesized NPs. The synthesized ZnO NPs have shown antibacterial efficacy against Gram-positive and Gram-negative pathogens. Combined effects of ZnO NPs and streptomycin showed increased efficacy or zone of clearance as compared to the efficacy of either ZnO NPs or streptomycin alone. Therefore, these ZnO NPs may be used as chemotherapeutic or preventive agents against the pathogens. The synergism of ZnO NPs and common antibiotic agents could be beneficial in the formulation of antibacterial products for their use in agriculture, pharmaceutical industries, cosmetics as well as for preparing nano-medicines.

## Author Contributions

MG done the all the experiments and wrote the manuscript draft. RT and SK proof edit the initial draft. RM and DS design the concept and finalized the manuscript.

## Conflict of Interest Statement

The authors declare that the research was conducted in the absence of any commercial or financial relationships that could be construed as a potential conflict of interest. The handling Editor declared a shared affiliation, though no other collaboration with one of the authors DS.
